# Volatile versus propofol sedation after cardiac valve surgery: a single-center prospective randomized controlled trial

**DOI:** 10.1186/s13054-024-04899-y

**Published:** 2024-04-05

**Authors:** Armin Niklas Flinspach, Florian Jürgen Raimann, Philipp Kaiser, Michaela Pfaff, Kai Zacharowski, Vanessa Neef, Elisabeth Hannah Adam

**Affiliations:** 1 Department of Anaesthesiology, Intensive Care Medicine and Pain Therapy, University Hospital Frankfurt, Goethe-University Frankfurt, Theodor-Stern Kai 7, 60590 Frankfurt am Main, Germany; 2 Department of Cardiothoracic Surgery, University Hospital Frankfurt, Goethe-University Frankfurt, Frankfurt am Main, Germany

**Keywords:** Cardiac surgery, Volatile sedation, Management, Awakening, Cardiac Valve Prosthesis, Critical care

## Abstract

**Background:**

Optimal intensive care of patients undergoing valve surgery is a complex balancing act between sedation for monitoring and timely postoperative awakening. It remains unclear, if these requirements can be fulfilled by volatile sedations in intensive care medicine in an efficient manner. Therefore, this study aimed to assess the time to extubation and secondary the workload required.

**Methods:**

We conducted a prospective randomized single-center trial at a tertiary university hospital to evaluate the postoperative management of open valve surgery patients. The study was randomized with regard to the use of volatile sedation compared to propofol sedation. Sedation was discontinued 60 min after admission for critical postoperative monitoring.

**Results:**

We observed a significantly earlier extubation (91 ± 39 min vs. 167 ± 77 min; *p* < 0.001), eye-opening (86 ± 28 min vs. 151 ± 71 min; *p* < 0.001) and command compliance (93 ± 38 min vs. 164 ± 75 min; *p* < 0.001) using volatile sedation, which in turn was associated with a significantly increased workload of a median of 9:56 min (± 4:16 min) set-up time. We did not observe any differences in complications. Cardiopulmonary bypass time did not differ between the groups 101 (IQR 81; 113) versus 112 (IQR 79; 136) minutes *p* = 0.36.

**Conclusions:**

Using volatile sedation is associated with few minutes additional workload in assembling and enables a significantly accelerated evaluation of vulnerable patient groups. Volatile sedation has considerable advantages and emerges as a safe sedation technique in our vulnerable study population.

*Trial registration*: Clinical trials registration (NCT04958668) was completed on 1 July 2021.

## Background

Intensive care medicine is vital in managing patients after heart valve surgery. Heart valve reconstruction and replacement are complex and invasive procedures requiring extensive monitoring to assure clinical stabilization [1]. During this time of recovery, deep sedation is frequently employed [2, 3]. However, prolonged sedation risks negative sequelae that potentially impact the patient's recovery and overall health. Consequently, fast-track strategies promoting extubation within six hours after intensive care unit (ICU) admission have emerged as the normative practice [4–6]. Notably, prolonged sedation correlates with increased incidence of delirium, contributing to protracted ICU stays, increased mortality rates and postoperative cognitive deficits [7–9]. Prolonged sedation and ventilation predisposes patients to muscle atrophy, possibly culminating in ICU-acquired weakness (ICUAW) or an elevated risk of ventilator-associated pneumonia [10–12]. Earlier and predictable awakening with an equally accelerated neurological assessment may also help to detect neurological complications requiring intervention at an early stage. These periprocedural risks underscore the importance of closely monitoring patients to limit the duration of sedation and ventilation [1, 13, 14].

The increase in the number of patients requiring surveillance, alongside a rise in comorbid conditions with the associated surge in nursing workload, also needs to be considered for practicability in the implementation of novel techniques. Different sedation procedures are associated with different personnel and cost expenses, some of which appear to be amenable to bedside evaluation.

The use of sedatives in economically constrained health care systems must be carefully considered and balanced against the potential risks and benefits.

For the aforementioned reasons, shortening sedation should be considered in patients, e.g., by the use of volatile sedatives. While semiclosed circuit ventilators predominate in surgical settings, open circuit ventilators are used exclusively in intensive care. This difficulty prompted the development and deployment of anesthetic conserving devices (ACDs), facilitating the conservation and re-administration of volatile anesthetics after their application into the airway [15, 16]. This method enabled the widespread adoption of volatile sedatives in the ICU. Their application in complex patient cohorts, including those with renal and hepatic insufficiency or obesity, poses minimal challenges, albeit with noted contraindications in patients with pre-existing obstructive pulmonary diseases. The favorable properties of volatile sedatives, characterized by minimal adverse effects, rapid awakening facilitating earlier extubation, and significant bronchodilatory and anti-inflammatory properties, renders them near-ideal agents for sedation, significantly reducing ventilation time [17–21]. The discourse surrounding the potential diminution of delirium incidence after deep sedation persists, with evidence not consistently indicating delirium rate reductions [22]. Volatile anesthetics offer dosage scalability without the habituation or limitations seen with propofol, beneficial in challenging sedation cases [23, 24]. With respect to the widely used substance propofol, a risk of propofol infusion syndrome (PRIS) has been reported [25]. While the incidence of PRIS is slightly greater than 1%, the use of volatile sedatives carries a genetically predisposed risk of malignant hyperthermia (MH), with an occurrence rate ranging from 1 in 10,000 to 1 in 250,000 anesthetic administrations [26]. Although the critical nature of both conditions, the effective management of MH with dantrolene has reduced mortality rates to 2 and 6%, compared to the higher mortality associated with PRIS [27, 28]. The impact of necessary equipment on personnel resources remains uncertain, with studies indicating low substance consumption over extended periods, yet lacking clear data for short term usage [29–32]. As an approach to clarify the alleged additional effort, we conducted a study to evaluate the advantages of corresponding sedation procedures with an assessment of the workload in a cohort of patients undergoing valve surgery. The primary study outcome focused on the time to extubation, while secondary outcomes encompasses the workload required for sedation management, course of the surgical procedure, neurocognitive recovery time, setup duration, incidence of acute renal failure, and catecholamine usage, among others.

## Methods

### Trial design

We conducted a prospective, randomized clinical trial at a tertiary university hospital. The protocol was approved by the institutional ethics committee (#20-1050) and registered at clinicaltrials.gov on the 1st of July 2021 (NCT04958668) preceding the enrolment of the inaugural patient. Informed consent was obtained from each individual patient or their legal representative in advance. The study’s protocol, designed in strict accordance with the recommendations of the Declaration of Helsinki, has previously been published [33, 34]. The manuscript adheres to the Consolidated Standards of Reporting Trials (CONSORT) guidelines [35].

### Randomization

Randomization was performed using block randomization generated by the consulting biostatisticians affiliated with the authors’ institution prior to the study’s initiation. Following the valve surgery, block randomization was disclosed to allocate the treatment groups and to perform the corresponding sedation technique.

### Participants

Within the study period from November 1, 2021, to August 15, 2023, all cardiac surgical procedures conducted were subject to evaluation for inclusion. Eligibility criteria were applied to potential patients undergoing valve surgery. Patients subjected to emergency surgical interventions—for example, during the night or weekends—were not included in the study due to the absence of prospective informed consent [33]. Patients were included until the number of cases calculated prior to the study (n = 50 patients per study group) was reached.

### Consent

Eligible patients were approached for consent, which was obtained after bedside evaluation and subsequent written informed consent. After providing the standard anesthesiological and surgical informed consent, patients were verbally briefed on the study by a physician on the evening prior to the scheduled surgery. Upon agreeing to participate, their consent was formally documented in writing on the provided consent form.

Study participation was predicated on age (> 18 years), ICU admission following heart valve surgery, and prior informed consent. Exclusion criteria included known intolerance to volatile anesthetics (e.g., MH), severe obstructive pulmonary disease, concurrent major aortic surgery, or unexpected severe complications (e.g., necessitating extracorporeal life support).

The bedside setup for eligible patients included, special equipment, notably an ACD, alongside essential monitoring equipment to administer volatile sedation. Ecological considerations and workplace safety protocols mandated the capture of respiratory exhaust, utilizing filter systems (CONTRAfluran™, Zeosys Medical GmbH, Luckenwalde, Germany), to absorb exhaled volatile anesthetics, thereby ensuring environmental and occupational health compliance.

### Study protocol

Detailed prospective descriptions of the treatment modalities have been published previously [33]. All patients included in the study received controlled mechanical ventilation and endotracheal intubation according to the department's standardized treatment protocols. Intraoperative anesthesia prior to ICU admission was performed in both study arms in a consistent, standardized manner using propofol in combination with initial fractionated administration of 1 mg of fentanyl followed by the short-acting context-insensitive opioid remifentanil.

### Interventions

Upon admission to the ICU, sedation was guided by block randomization to block randomization to either propofol or sevoflurane using an ACD. Both the commercially available AnaConDa^®^ (Sedana Medical AB, Danderyd, Schweden) and MIRUS^®^ (Technologie Institut Medizin GmbH, Tübingen, Germany) systems were used to administer sevoflurane. Bed unit preparation and, when required, volatile sedation initiation times were recorded on paper-based study case report forms (CRF). Following the surgical procedure, intensive care therapy was provided according to the in-house standard operating procedure (SOP) for valve surgery, maintaining sedation depth in both groups at a Richmond Agitation and Sedation Scale (RASS) of − 3 to − 4. Respiratory weaning adhered to intrahospital guidelines during the sedation phase. The patient’s condition was assessed at 60 min after admission to the ICU to determine whether sedation could be discontinued. This evaluation was conducted using a protocol implemented in the paper based CRF. The criteria assessed included postoperative bleeding, circulatory and respiratory stability. Further therapy was based on the discretion of the critical care specialist, who was responsible for the protocol-based start, adjustment if necessary, and discontinuation of the sedatives. If the patient's condition was stable according to the predefined criteria in the study protocol (cardio-respiratory stability, absence of lactate accumulation, adequate body temperature, and no signs of bleeding) 60 min after admission, sedation was terminated. Evaluations of the time required for spontaneous breathing, hand grasping on demand, eye opening, and extubation was conducted for study purposes. Consumption of medical supplies (syringes, feeding syringe lines, heat-moisture exchange filters, and ACD) and pharmaceuticals for sedation and cardiovascular support was quantified, as well as the amount of work involving in dismantling volatile sedative delivery and absorber systems.

### Data collection

Clinical data were systematically recorded using a patient data management system (PDMS; Metavision 5.4, iMDsoft, Tel Aviv, Israel). The comprehensive study documentation on the CRF encompassed patient demographics, laboratory analyses, ventilation parameters, specific laboratory markers indicative of inflammatory response, renal, hepatic, and cardiac function. Additionally, metrics such as time to wakefulness, time to extubation, and overall workload were recorded by trained study personnel who remained distinct from the clinical treatment team. The time allocated for routine bedside preparation, entailing medication organization, equipment configuration (e.g., suction and ventilator circuits), and preliminary technical checks (e.g., leak tests), was rigorously recorded. Furthermore, the additional time allocated for establishing the technical conditions and ACD prerequisites for volatile sedation was also recorded (time for volatile sedation preparation). Likewise, the awakening process and the duration until wakefulness were monitored by trained study staff at the bedside using a CRF. This approach also extended to the precise tallying of consumed materials and pharmacological substances. Screening for potential complications, including severe vasoplegia, postoperative hemorrhage, acute renal failure, liver dysfunction, cerebrovascular incidents, emerging neurocognitive impairment, and delirium adhered to established ICU protocols. Delirium assessment of the Confusion Assessment Method for the Intensive Care Unit (CAM-ICU) applied after awakening and at the commencement of each nursing shift, thus every 8 h [36].

### Statistical methods

Consistent with the published study protocol, sample size calculations were performed. Given an effect size of 0.5, an α-error probability of 0.05, and a power of 0.80 the total sample size for the 2 study groups was 33 patients per study group. Fifty patients were included in each study arm to compensate for missing values or deviations from the exponential distribution assumption.

The categorical variables are presented as counts and percentages. Variables that were not normally distributed are described as medians (interquartile range, IQR 25/75). Demographics and clinical differences between groups were assessed using Fisher’s exact test for categorical variables and the Mann–Whitney U-test and the Kruskal–Wallis test for continuous variables, as appropriate.

All the statistical tests were two-tailed, and results with *p* < 0.05 were considered to indicate statistical significance. All analyses were performed with SPSS^®^ (IBM Corp., Version 29, Chicago, IL, USA).

## Results

**Fig. 1:**
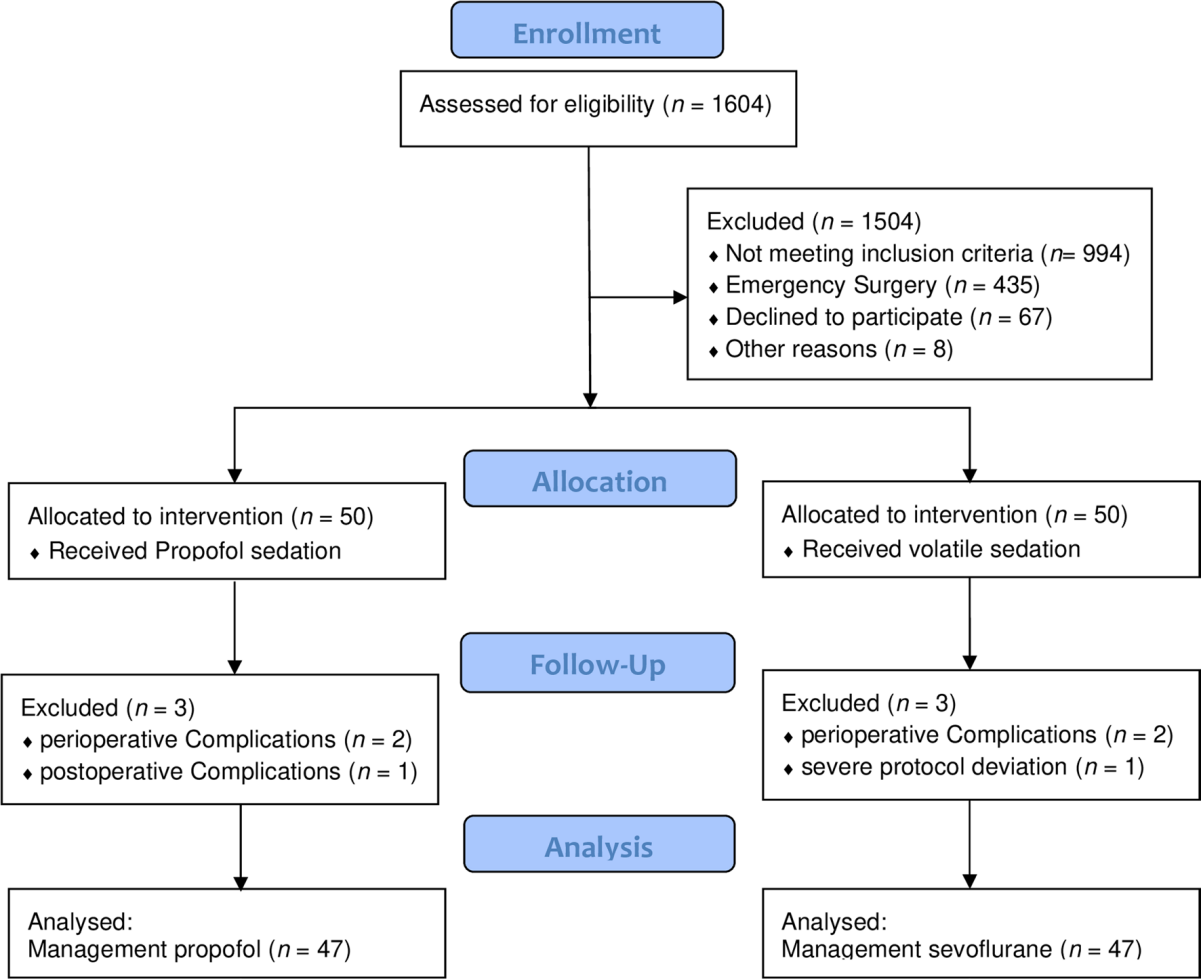
CONSORT diagram of prospective patient inclusion. Flow chart of the screening process and study inclusion, indicating reasons for exclusion, following the CONSORT guideline. ^≠^No protocol-based evaluation regarding the end of sedation within the time frame

Among 1604 patients screened, 175 met eligibility criteria. Written informed consent was obtained from 100 patients. Within the intended study period, six patients were excluded, with specifics detailed in Fig. 1.

Accordingly, 47 patients were treated with propofol in the postoperative phase, while 47 patients were sedated with sevoflurane. Patient characteristics are presented in Table 1.

**Table 1:** Overview of the patient’s characteristics

	Volatile	Intravenous	All
*n* =	47 (50%)	47 (50%)	94 (100%)
Sex (male, %)	33 (70.2%)	33 (70.2%)	66 (70.2%)
Age (years)	60 (IQR 51; 69)	64 (IQR 57; 71)	62 (IQR 54; 71)
Diabetes mellitus	9 (19.1%)	6 (12.8%)	15 (16.0%)
Hyperlipidemia	19 (40.4%)	15 (31.9%)	34 (36.2%)
Chronic renal failure	3 (6.4%)	6 (12.8%)	9 (9.6%)
Arterial hypertension	36 (76.6%)	34(72.3%)	70 (74.5%)
Smoking	6 (12.8%)	7 (14.8%)	13 (13.8%)
BMI (kg/m^2^)	26.3 (IQR 23.4; 29.0)	25.0 (IQR 23.0; 28.7)	25.8 (IQR 23.3; 28.7)
Euro II Score	1.18 (IQR 0.78; 1.84)	1.30 (IQR 0.88; 2.13)	1.24 (IQR 0.81; 2.07)
Mitral valve surgery	25 (53.2%)	25 (53.2%)	50 (53.2%)
Tricuspid valve surgery	3(6.4%)	5(10.6%)	8 (8.5%)
Aortic valve surgery	22 (46.8%)	21 (44.7%)	43 (45.7%)
Combined Surgery	3 (6.4%)	4 (8.5%)	7 (7.4%)

Clinical characteristics of the patients included in the study correspond to the sedation intervention groups and in total. Data are presented as median with interquartile range (IQR) or as patient number (percentage) where applicable

kg, kilogram; m^2^, square meters

Our analysis revealed no significant differences between the study groups in terms of surgical procedures, demographics, pre-existing medical conditions, or the EuroSCORE II risk stratification [37]. The laboratory assessments conducted six hours after admission indicated a significant difference in creatinine levels, with a corresponding increase within the propofol group, albeit without a corresponding increase in acute renal failure incidents. Furthermore, a significant decrease in the interleukin 6 level was observed six hours after ICU admission. A differential analysis of the evaluated clinical and laboratory treatment parameters is delineated in Table 2.

**Table 2:** Therapy characteristics and laboratory results

	Volatile	Intravenous	*p* value
Incision to suture (min)	193 (IQR 158; 219)	211 (IQR 159; 246)	0.296
CPB (min)	101 (IQR 81; 113)	112 (IQR 79; 136)	0.360
Aortic clamping (min)	60 (IQR 53; 73)	62 (IQR 53; 75)	0.454
Operative anesthesia* (min)	288 (IQR 250; 334)	290 (IQR 264; 364)	0.126
*Laboratory results*			
Creatinine 0 h^≠^ (mg/dl)	1.03 (IQR 0.72; 1.14)	1.06 (IQR 0.79; 1.09)	0.081
Creatinine 6 h^≠^ (mg/dl)	1.08 (IQR 0.76; 1.12)	1.14 (IQR 0.83; 1.12)	0.044
Troponin T 0 h^≠^ (pg/ml)	827 (IQR 268; 1021)	683 (IQR 307; 753)	0.904
Troponin T 6 h^≠^ (pg/ml)	1,159 (IQR 452; 1432)	546 (IQR 404; 871)	0.591
CK-MB 0 h^≠^ (mg/dl)	53.0 (IQR 38; 88)	48.0 (IQR 37; 68)	0.752
CK-MB 6 h^≠^ (mg/dl)	54.0 (IQR 35; 87)	47.0 (IQR 33; 63)	0.366
CK-MB 18 h^≠^ (mg/dl)	45.0 (IQR 30; 75)	34.0 (IQR 27; 59)	0.245
Interleukin six 0 h^≠^ (pg/ml)	179 (IQR 89; 322)	235 (IQR 98; 547)	0.063
Interleukin six 6 h^≠^ (pg/ml)	134 (IQR 106; 264)	193 (IQR 104; 306)	0.035
Interleukin six 18 h^≠^ (pg/ml)	103 (IQR 83; 190)	135 (IQR 87; 226)	0.669

Table of clinical and laboratory treatment parameters:

CK-MB, creatine kinase-myocardial band; CPB, cardiopulmonary bypass; postOP, postoperative; h, Hour; mg, milligram; dl, deciliters, pg, picogram; ml milliliters; min, minute

^≠^Time after admission to the ICU

*Start of anesthesia induction to arrival on ICU

The median preparation time for sevoflurane sedation was 26:31 min (IQR 23:38; 31:54) compared to 18:35 min (IQR 13:23; 23:19) for propofol-based sedation, indicating a significant additional median preparation time of 9:56 min (± 4:16 min) for additional (*p* < 0.001). During the sedation phase, sufficient sedation (RASS -3/-4) was achieved at a MAC_50_ of 0.7 (± 0.1), with no significant need for additional sedatives to attain sufficient sedation depth. The corresponding average consumption of propofol was 841 mg ± 1498 mg, while for sevoflurane, it was 14 ± 4 ml.

**Fig. 2:**
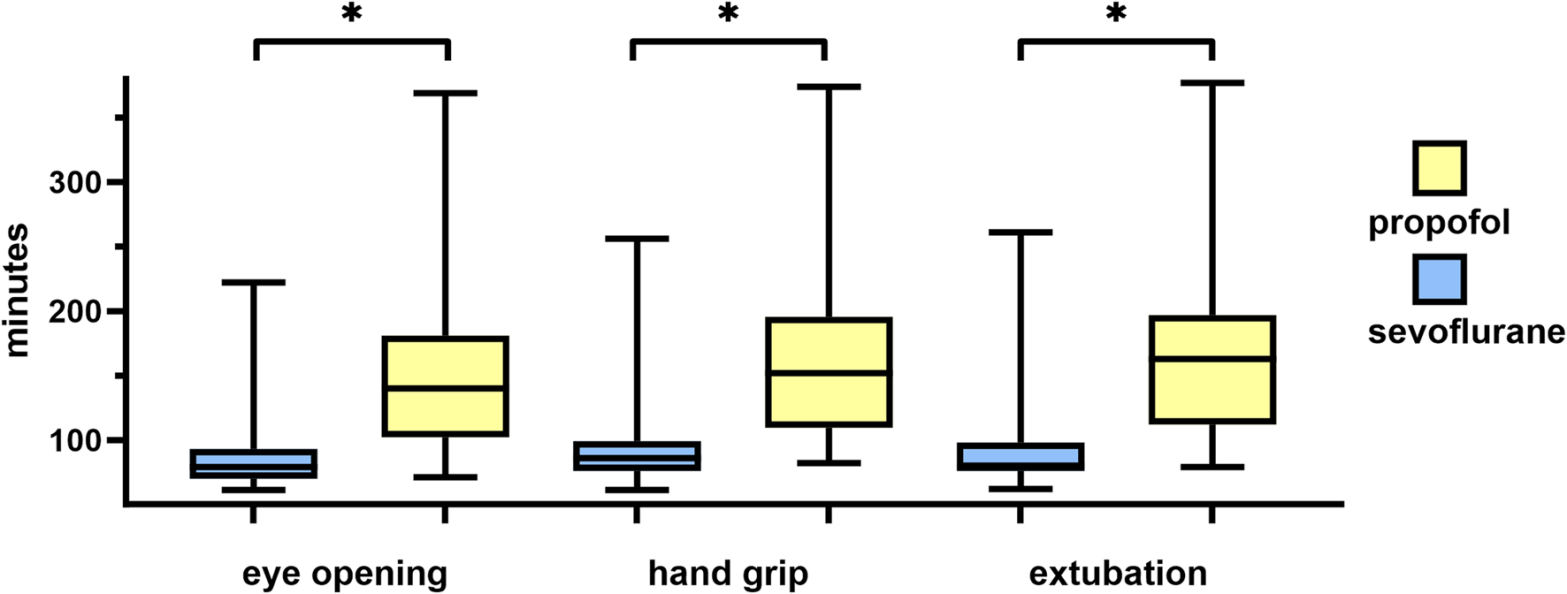
time required for responses. Box-plot bar chart of the time comparison of the study groups regarding the recorded time to eye opening, intentional motor response and extubation

Sevoflurane treatment was associated with significantly reduced times until eye opening (86 ± 28 min versus 151 ± 71 min; *p* < 0.001), command compliance (hand grip) (93 ± 38 min versus 164 ± 75 min; *p* < 0.001) and extubation (91 ± 39 min versus 167 ± 77 min; *p* < 0.001) following cessation of postsurgical sedation, as detailed in Fig. 2.

No significant difference in the incidence of delirium on the first postoperative day was observed between the two groups (propofol, *n* = 3, 6.4% versus sevoflurane, *n* = 4, 8.5%, *p* = 0.665) or on the third postoperative day (*n* = 4, 8.5% vs. *n* = 8, 17.0%), *p* = 0.416.

The time to discharge to the normal ward averaged 21 h 28 min (± 10 h 48 min) for sevoflurane versus 29 h 58 min (± 20 h) for propofol, with the difference not reaching statistical significance (*p* = 0.078).

In terms of catecholamine usage for circulatory stabilization during deep sedation, no significant differences were observed in norepinephrine dosages between treatments within the first 60 min in the ICU: 485 ± 408 µg for sevoflurane treatment and 735 ± 1876 µg for propofol treatment. The numbers of patients requiring the antidiuretic hormone analogue vasopressin (*n* = 7 versus 1, *p* = 0.007), as well as epinephrine (*n* = 11 versus 4, *p* = 0.033), were significantly higher under propofol sedation.

Throughout the monitoring of potentially adverse effects, oxygenation levels (*p* = 0.710) or ventilation parameters exhibited no significant variances, and there was no severe lactate accumulation. According to international diagnostic criteria, nine patients in the propofol group experienced acute renal failure compared with four in the volatile sedation group (*p* = 0.149); all resolving without intervention or renal replacement therapy. Postoperative nausea and vomiting were noted once in each treatment group. We did not observe any liver failure in the study cohort, and there were no in-house fatalities reported across both groups.

## Discussion

In our cohort of open cardiac valve surgery patients who underwent cardiopulmonary bypass intraoperatively, a significant time advantage with regard to the ability to extubate under short-term sedation was observed, corroborating the findings of prior research [17, 19]. After 60 min of surveillance, the time to extubation was notably shorter for sevoflurane (31 min), compared to propofol-induced sedation (107 min). This aligns with several comprehensive analyses of published studies on the utilization of volatile sedatives in ICU environments, which have highlighted the effectiveness of agents such as isoflurane and sevoflurane across varied patient populations [19, 38, 39]. A salient point of convergence among these studies is the observed significant reduction in time to extubation, a result that aligns closely with our own observations. Furthermore, our study allowed us to quantify the time required to set up and dismantle a corresponding ACD system for bedside application in the ICU.

We were able to show that a minimal technical effort of less than ten minutes enabled a significant acceleration of the awakening, exceeding one hour, thus permitting an earlier assessment of the patient's neurological condition. The increased technical effort seamlessly integrated into clinical workflows, proving its non-disruptive and delegable nature, and was well accepted by the ICU staff, including physicians, nurses, and surgeons.

The consumption rates of propofol and sevoflurane observed, were surprisingly higher than those previously reported, particularly for sevoflurane, where we identified an average usage of 14 milliliters, significantly surpassing the approximately 8 ml described in extended ICU sedation scenarios [15, 30, 40–43]. This discrepancy might be attributed to the initial filling of the ACD and the saturation process of the patient's physiological compartments.

Regarding the safety profile of sevoflurane, no complications were observed during the study. The exclusions were primarily attributed to intraoperative complications or postoperative surgical bleeding. This underscores the feasibility and safety of the short-term sevoflurane sedation in cardiothoracic patients, in line accordance with previously published research [17]. Concerns regarding renal damage from elevated serum fluoride levels with the use of sevoflurane were not substantiated by our laboratory findings [44, 45]. Additionally, diverging from other studies, an increased need for catecholamines was not detected when using sevoflurane in this susceptible patient group, aligning with the cardiovascular stability observed in patients subjected to prolonged volatile sedation [44]. Conversely, propofol sedation significantly increased the necessity for vasopressin and epinephrine [46, 47], a phenomenon likely attributable to propofol’s established vasodilatory effects.

Consequently, vasodilatory effects might have been absent under deep sedation induced by midazolam; yet, the application of benzodiazepines could have notably prolonged the awakening period [48, 49].

Although we did not observe any intraprocedural cerebrovascular incidents within our cohort, such events remain a relevant risk, manageable by early detection and timely intervention. The accelerated ability to extubate patients and the consequent swift neurological assessment could diminish the delay to conduct diagnostic imaging for neuropathological identification and facilitating appropriate treatment. This principle extends to the management of unexpected coronary perfusion malfunctions. In addition to these specific valve surgery aspects, the broader impact of the link between delirium and both the length and depth of postoperative sedation warrants attention.

Economic aspects, especially the substantial investment costs for the ACD, repeatedly deter the adoption of volatile sedation in the ICU. Nonetheless, the well-documented clinical benefits of inhaled sedation are likely to justify the initial outlay and minimal additional personnel effort outweighing these concerns in the long term.

A significant decrease in interleukin 6 levels, identified within in the volatile sedation group, corresponds with the previously described anti-inflammatory effects of these sedatives. The direct benefits of mitigating proinflammatory responses, have not been investigated in the context of postoperative rehabilitation, highlighting the imperative for further exploration of this class of agents for long-term intensive care sedation.

We believe that our results are likely to be transferable to other patient groups and may demonstrate comparable benefits in terms of controllability, a minimal safety profile, and rapid awakening.

This study has several limitations. Conducted at a single center without the possibility of practitioner blinding, the research design could introduce selective sedative use, potentially skewing outcomes. Our research center had pre-existing expertise administering volatile sedation using both AnaConDa and MIRUS ACD systems, thus requiring minimal additional training for staff. Conversely, institutions adopting to ACD-based sedation techniques a new may encounter prolonged times for setup and dismantling periods for bedside functionality. Conducting a multicenter trial would be advantageous to comprehensively assess these variations. Moreover, limiting participation to individuals scheduled for elective heart valve surgery, consequently reduced the study’s generalizability to a wider spectrum of cardiac surgery patients.

Due to the short duration of sedation and the consecutive short duration of ventilation and ICU stay, we were unable to determine whether pneumonia occurred during the course of the treatment. Furthermore, as a pilot study with a limited study population, certain findings might have been more definitive with a larger study cohort.

## Conclusions

The short-term application of volatile sedatives for intensive care sedation is associated with additional work and technical effort for the ICU staff while also significantly accelerating the wake-up response and neurological assessability following cardiothoracic valve surgery.

## Data Availability

The data cannot be shared publicly. The datasets generated and/or analyzed during the current study are not publicly available due to national data protection laws but are available upon reasonable request from the corresponding author or via the data protection officer of the University Hospital of Frankfurt [Datenschutz@kgu.de (www.kgu.de, accessed on 24 September 2023)].

## Abbreviations

ACD: Anesthetic conserving device
BMI: Body mass index
CAM-ICU: Confusion Assessment Method for the Intensive Care Unit
CK-MB: Creatine kinase-myocardial band
CONSORT: Consolidated Standards of Reporting Trials
COVID-19: Coronavirus Disease 2019
CPB: Cardiopulmonary bypass
IQR: Interquartile range
ICU: Intensive Care Unit
ICUAW: ICU acquired weakness
MAC: Minimal alveolar concentration
PDMS: Patient data management system
PICS: Postintensive care syndrome
PRIS: Propofol infusion syndrome
RASS: Richmond agitation and sedation scale
SOP: Standard operating procedure
